# The rise of AI in the workplace: why perceived organizational support fails to protect long-term career satisfaction

**DOI:** 10.3389/frai.2026.1891537

**Published:** 2026-09-18

**Authors:** João Matias, Joana Carneiro Pinto

**Affiliations:** Universidade Católica Portuguesa, Faculdade de Ciências Humanas CEDH - Centro de Investigação para o Desenvolvimento Humano, Lisbon, Portugal

**Keywords:** AI-related insecurity, artificial intelligence, career competency, career satisfaction, job security, organizational support

## Abstract

**Introduction:**

The growing integration of Artificial Intelligence (AI) into the workplace is reshaping employees’ perceptions of job and career stability, potentially generating psychological stress and uncertainty about future career trajectories. Drawing on Job Demands–Resources (JD–R) Theory, this study examines the relationship between AI-related insecurity and three career outcomes—job security, career competency, and career satisfaction—and investigates whether Perceived Organizational Support (POS) moderates these relationships.

**Methods:**

A quantitative-dominant mixed-methods design was employed with a sample of 106 Portuguese professionals. Quantitative analyses examined the associations between AI-related insecurity and the three career outcomes and tested the moderating role of POS. Supplementary qualitative data were used to provide contextual insight into employees’ experiences and perceptions of organizational responses to AI-related workplace change.

**Results:**

AI-related insecurity was significantly and negatively associated with perceived job security and career satisfaction, whereas its association with career competency was not statistically significant. Moderation analyses indicated a differentiated role of POS across career outcomes. Higher POS attenuated the negative relationship between AI-related insecurity and job security but did not significantly moderate its relationship with career satisfaction. Qualitative findings further suggested that limited organizational communication and insufficient opportunities for upskilling may intensify employees’ uncertainty and anxiety regarding AI-driven workplace change.

**Discussion:**

The findings suggest that organizational support may help employees manage immediate concerns about job continuity associated with AI integration but may be less effective in addressing broader uncertainty about long-term career trajectories. This distinction highlights the limits of generalized organizational support as a response to AI-related career insecurity and points to the importance of combining supportive organizational practices with transparent communication, targeted upskilling, and career development initiatives. The study extends the application of JD–R Theory to AI-related workplace change by showing that organizational resources may have differential protective effects across distinct dimensions of career insecurity.

## Introduction

Artificial Intelligence (AI) and generative technologies are fundamentally and irreversibly transforming global productivity paradigms and organizational management frameworks (Schwab, 2017). While organizational adoption is largely driven by the imperative for competitive advantage and working efficiency (Alsheibani et al., 2020; Chalmers et al., 2026), this rapid technological evolution inherently generates significant psychological and structural disruption for the human workforce (Magliocca et al., 2025; Rasool et al., 2022). This research aims to explore the complex human-technology interface, focusing on Perceived Organizational Support (POS) as a crucial resource that shapes employee adaptation during AI implementation processes in their work contexts. More specifically, this study proposes the development of a comprehensive theoretical framework (see Figure 1) that considers POS’s influence as a moderating resource in mitigating psychological stress associated with AI-related insecurity across three important dimensions of professional well-being: Job Security, Career Competency, and Career Satisfaction.

**Figure 1:**
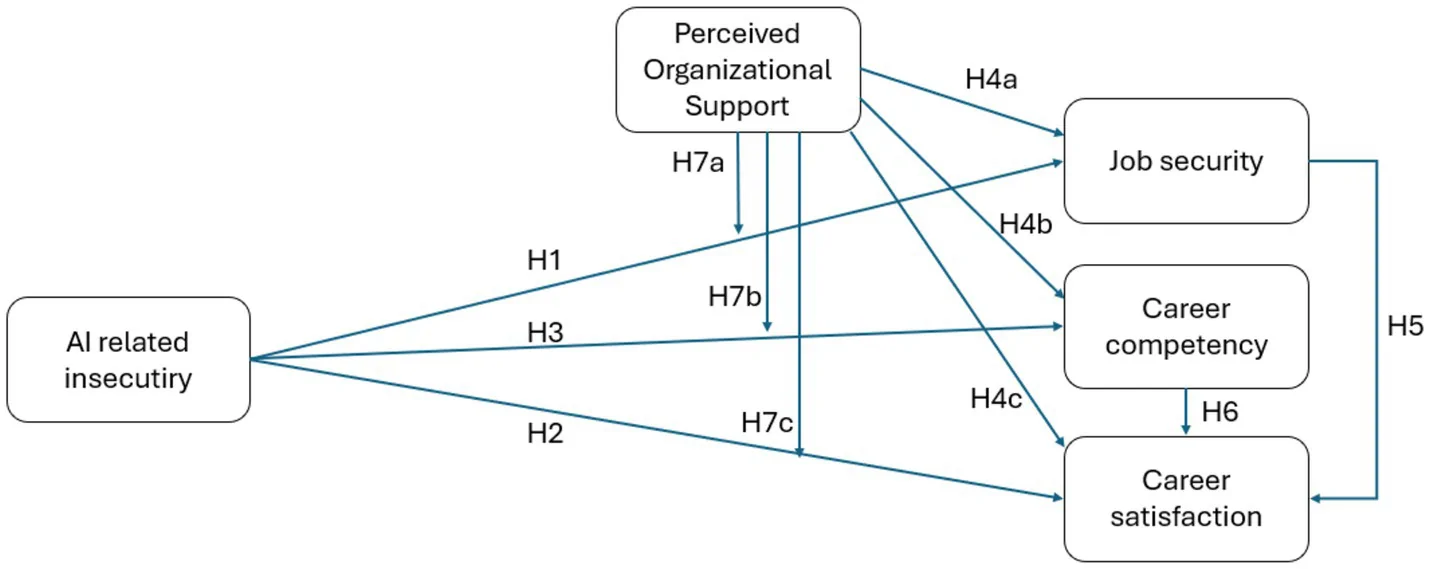
Theoretical framework. While AI awareness serves as the situational precursor, this study specifically measures AI-related insecurity as the psychological demand impacting employee outcomes.

The focus on Portuguese organizational environments contributes valuable empirical *insights* from Southern European contexts, where the literature on technological transition remains significantly underexplored. Most existing literature focuses on highly automated Anglo-Saxon market realities, leaving a notable gap regarding how professionals in Southern European economies—which often exhibit distinct organizational cultures and different paces of digital transition-perceive and adapt to automated work environment shifts.

To fully understand this transition, it is essential to ground it not merely as a technical update, but within a broader sociotechnical systems framework (Makarius et al., 2020). Sociotechnical theory posits that any organizational intervention consists of two intertwined systems: the technical (the AI tools and algorithms) and the social (the human relationships, psychological safety, and roles). A failure to balance both systems often results in structural misalignment and psychological strain. Furthermore, the introduction of AI must be viewed through the lens of AI ethics in the workplace. Ethical AI integration extends beyond data privacy or algorithmic fairness; it is fundamentally a workplace health and safety issue that impacts employee dignity, professional autonomy, and psychological well-being (Cebulla et al., 2022; Salvi del Pero et al., 2022). When organizations implement AI without clear ethical frameworks or proactive change management, it risks eroding the social fabric of the workplace and inducing persistent professional insecurity.

The overarching aim of this study is to investigate the complex psychological dynamics at the human-technology interface by exploring how AI-driven job demands relate to critical indicators of long-term career well-being. Specifically, we seek to understand the extent to which generalized organizational resources can successfully shield employees from the unique, multi-layered anxieties induced by rapid technological disruption. The specific, testable hypotheses derived from this conceptual goal are detailed sequentially in the following section.

### Theoretical framework and hypothesis

The theoretical framework underpinning this study is the Job Demands-Resources (JD-R) Theory (Bakker and Demerouti, 2017). This model is suitable for analyzing technological disruption by conceptualizing workplace factors as either Job Demands-stressors requiring sustained effort and leading to strain-or Job Resources-factors promoting development and mitigating the impact of demands. In the model proposed in this article, AI-related Insecurity is framed as the primary psychological demand (a hindrance demand), while POS functions as the core organizational resource (Eisenberger et al., 1986).

Before delineating specific relationships, it is necessary to define the core constructs. AI-related insecurity is operationalized as an employee’s subjective, multi-dimensional perception of threat regarding the potential of automated technologies to disrupt their career stability and diminish the future relevance of their professional skill sets (Brougham and Haar, 2018; Kong et al., 2021). Perceived Organizational Support (POS), conversely, represents the generalized global belief held by employees regarding the extent to which their organization values their work contributions and genuinely cares about their personal well-being (Eisenberger et al., 1986).

When these two constructs interact, a complex phenomenon termed “asymmetric buffering” may occur. Traditional JD-R models often assume that a strong job resource can uniformly weaken the negative impact of a job demand across all outcomes. However, rapid technological shifts like AI implementation induce distinct tiers of anxiety: proximate, tangible fears (e.g., immediate job loss) and distal, existential career anxieties (e.g., identity loss or skill obsolescence). Asymmetric buffering suggests that while generic organizational support effectively neutralizes the proximate, concrete threat of immediate job displacement, it may remain fundamentally insufficient to alleviate the deeper, strategic, and more intangible anxieties regarding long-term career trajectory and future relevance.

AI-related insecurity is presently conceptualized as a workplace-specific phenomenon: the employee’s subjective perception of the threat of AI’s potential to impact their career trajectory and the future relevance of their skills (Brougham and Haar, 2018; Kong et al., 2021; Lin and He, 2024). This perception stems from the employee’s analysis of the macroeconomic reality of automation, particularly concerning cognitive tasks (Acemoglu and Restrepo, 2018), which can generate psychological strain. Specifically, the perceived threat of substitution-both quantitative (fear of job loss) and qualitative (fear of one’s role in the company becoming obsolete) is hypothesized to be a negative predictor of professional insecurity (Sadeghi, 2024; Zhou et al., 2024). This conceptualization aligns the variable with the Hindrance Demands category of the JD-R model, which directly leads to strain and negative outcomes. This tension results in significant emotional fatigue among employees, intensifying feelings of job insecurity and general psychological stress (Teng et al., 2023). Previous empirical studies confirm these relationships. For example, a study by Hamid et al. (2024), developed in the hospitality sector, demonstrated that increased awareness of AI capabilities led to a significant reduction in job engagement, with job insecurity serving as the primary mediating variable for this adverse effect. Thus, we derive the following hypothesis:

*H1:* AI-related insecurity is negatively associated with job security.

Furthermore, this strain extends to Career Satisfaction, which consists of the employee’s overall subjective evaluation of their professional roles-specifically the degree of satisfaction, pleasure, and well-being they derive from performing their job, considering aspects such as autonomy and responsibility, learning potential, financial return, and organizational support, among others (Greenhaus et al., 1990; Kong et al., 2012; Spurk et al., 2011). Some studies, grounded in the cognitive transactional theory (Lazarus and Folkman, 1987), suggest a duality in the relationship between AI-related insecurity and Career Satisfaction, meaning AI can be appraised as a challenge or a hindrance (Xie et al., 2024). However, more recently, the escalating integration of AI has suggested an adverse effect on this outcome. This negative relationship can be theorized through both direct (lowered self-esteem; Ai, 2024) and indirect pathways (mediated by job burnout or decreased career self-efficacy; Abubakar, 2019; Kong et al., 2021; Li et al., 2025). The predominant psychological strain derived from technological impact establishes a strong theoretical rationale for a negative outcome.

*H2:* AI—related insecurity is negatively associated with career satisfaction.

Finally, the increasing sophistication of technology requires that organizations and their employees constantly reassess and adapt their Career Competencies (Bankins et al., 2024), which are key drivers of professional growth (Hirschi, 2012). Despite fears of obsolescence theoretically threatening Career Competency (Elfar, 2025; Makridakis, 2017), the relationship is nuanced. A study by Kong et al. (2021) found that AI-related insecurity did not directly affect employee competencies, but rather eroded organizational commitment, which, in turn, negatively impacted competency development. Conversely, a compelling counter-perspective frames AI as a tool for task augmentation. Under this view, employees maintain high self-efficacy by committing to adaptation and upskilling, thereby strengthening their perceived competence (Kong et al., 2021). The empirical test of this relationship is essential to resolve this theoretical ambiguity. Based on the theoretical risk of erosion and the documented indirect effects, we hypothesize:

*H3:* AI-related insecurity is negatively associated with career competency.

In contrast to technological demands, POS concerns employees’ perception that their organization values their contributions and is attentive to their well-being (Eisenberger et al., 1986; Rhoades and Eisenberger, 2002; Sun, 2019). During periods of crisis, such as AI integration, POS acts as a critical resource, providing both tangible resources (e.g., training, *reskilling* programs) and emotional resources (e.g., transparent communication, validation). This resource signals a reciprocal commitment, which is crucial for maintaining morale and promoting the motivation of all involved. Empirical evidence consistently supports the idea that robust POS positively predicts better professional outcomes (Stinglhamber and Caesens, 2020). By communicating investment, POS reinforces the belief in Job Security (Hellgren et al., 1999); motivates adaptive behavior, specifically the development of Career Competencies (Kong et al., 2021; Kuijpers and Scheerens, 2006); and ultimately drives Career Satisfaction (Greenhaus et al., 1990). Thus, we stipulate the following hypothesis:

*H4a, H4b, H4c:* POS is positively associated with job security, career competency, and career satisfaction.

To fully capture the path to overall Career Satisfaction, we incorporate the following sequential relationships between the model’s consequent variables. Job Security (Bella, 2023; Nielsen et al., 2017) serves as a fundamental “hygiene factor” (Herzberg, 1975) that prevents dissatisfaction (Alisic and Wiese, 2020; Nam, 2018). When employees perceive high security, it contributes positively to their career satisfaction, engagement, and commitment (Akpan, 2013; Domfeh and Hunsaker, 2020; Maryatmi, 2020; Papadimitriou et al., 2017).

*H5:* Job Security is positively associated with career satisfaction.

Furthermore, career competency (Akkermans et al., 2012; Kong et al., 2021; Kuijpers and Scheerens, 2006) acts as a “motivational factor.” Employees who perceive themselves as capable and in control of their professional growth are more likely to experience higher levels of career satisfaction (Akkermans et al., 2012; Kong et al., 2021). This positive relationship is well-established in the literature (Das and Chandrasekaran, 2022; Trivellas et al., 2015).

*H6:* Career competency is positively associated with Career satisfaction.

The final and most critical factor of this study is the test of the buffering hypothesis (Bakker and Demerouti, 2017; Kain and Jex, 2010). This central prediction postulates that resources (POS) will significantly weaken the negative influence of demands (AI-related insecurity) on employee outcomes (Kain and Jex, 2010). The literature demonstrates that high levels of POS can act as a psychological buffer against job insecurity (Bohle et al., 2018; Domfeh and Hunsaker, 2020; Wahyuni et al., 2020) and play a critical role in maintaining career satisfaction even in situations of disruption (Oubibi et al., 2022). Thus, we hypothesize that high levels of POS will effectively create a “psychological shield,” neutralizing the debilitating effects of technological anxiety and preserving stability and well-being across all measured career outcomes.

*H7a, H7b, H7c:* POS moderates the negative relationship between AI-related insecurity and job security, career competency, and career satisfaction.

This research aims to provide a critical contribution to the organizational literature by (1) Testing a complex moderation model that examines the efficacy of the POS *buffer* against the psychological threat posed by AI; (2) Offering novel, contemporary empirical data from the Portuguese context, enriching the dialogue on technological governance in Southern Europe; and (3) Producing essential information for practitioners, helping to design targeted HR policies that actively foster professional resilience.

## Methods

### Research design

This study adopts a quantitative-dominant cross-sectional research design, supplemented by qualitative insights from open-ended questions. This design is best categorized as an embedded qualitative approach, where qualitative text serves to contextually support and elaborate upon the primary statistical patterns discovered in the core regression analyses.

### Participants

Participated in this study 106 individuals, of which 74 women (69.8%) and 32 men (30.2%), aged between 19 to 77 years, and a mean age of 34.1 years (*SD* = 10.9). With respect to educational qualifications, 44.3% (*n* = 47) possessed a Master’s degree, 41.5% (*n* = 44) held a Bachelor’s degree, and 6.6% (*n* = 7) had completed a Ph.D. A minority, specifically 7.5% (*n* = 8) indicated that secondary education constituted their highest level of educational attainment. Regarding tenure in their current role, the most significant proportion of participants had been employed for a period ranging from 1 to 3 years (*n* = 46, 43.4%), followed by individuals with less than 1 year of employment (*n =* 24, 22.6%). Long-term employees were identified as those with tenures of 4 to 6 years (*n* = 16, 15.1%) and those exceeding 10 years (*n* = 14, 13.2%). A smaller percentage reported having a tenure between 7 to 10 years (*n* = 6, 5.7%).

The sample presented a professional diversity marked by the diversity of organizational type, organizational size, and economic sectors. Most of the participants worked for private enterprises (*n* = 81, 76.4%), whereas the rest were scattered between the public sector (*n* = 12, 11.3%), mixed ownership companies (*n* = 7, 6.6%), and nonprofit or cooperative organizations (*n* = 6, 5.7%). As for organizational size, most of the sample came from large firms (over 250 employees; *n* = 65, 61.3%), but other participants were also from medium (*n* = 16, 15.1%), small (*n* = 11, 10.4%) or micro enterprises (*n* = 14, 13.2%). The top economic sectors presented were the education and research (*n* = 30, 28.3%), media and entertainment (*n* = 13, 12.3%), commerce (*n* = 12, 11.3%), and information technology (*n* = 11, 10.4%). Smaller rates came from other activities, including healthcare, public administration, finance, and industry, thus confirming a wide professional background.

Sampling criteria were set such that this population would only include members from organizations which had previously had AI technology implemented. The time since implementation was not very distant: 57.5% (*n* = 61) reported that adoption had begun within the past year, and 32.1% (*n* = 34) within the past two years. Very few reported implementation from 3 to 5 years ago (*n* = 9, 8.5%) or over a decade ago (*n* = 2, 1.9%).

### Instruments

The assessment protocol consisted of a total of 46 questions, organized into five distinct instruments designed to evaluate the following dimensions: AI-related insecurity/ related insecurity, Perceived Organizational Support, Job Security, Career Competency, and Career Satisfaction.

While all measures were derived from previously validated instruments, their original items were systematically adapted to explicitly address the core context of AI transition and implementation in the workplace. The comprehensive nature of this adaptation yields an instrument that is methodologically innovative, ensuring its relevance for replication and establishing a new basis for both national and international research on AI’s impact.

To guarantee structural validity and reliability within our sample framework, psychometric metrics were formally evaluated via the calculation of Cronbach’s alpha (α), Composite Reliability (CR), and Average Variance Extracted (AVE) for each instrument following an Exploratory Factor Analysis (EFA). Discriminant validity was established utilizing the Fornell-Larcker criterion, ensuring that the square root of the AVE for each construct exceeded its highest correlation with any other variable in the model.

AI-related insecurity was measured using a four-item questionnaire developed by Kong et al. (2021), but re-labeled here to reflect the construct’s focus on personal concern and threat perception. Respondents completed the instrument using a 5-point Likert scale (1 = “Strongly disagree” to 5 = “Strongly agree”). The items assess personal concerns regarding the impact of AI on participants’ current employment and long-term professional prospects. A representative item is: “I am personally worried about my future in my industry due to AI potentially replacing employees.” For this study, all items were translated and culturally adapted into Portuguese. The scale demonstrated high internal consistency within the present sample (*α* = 0.88, CR = 0.89, AVE = 0.67).

Perceived organizational support (POS) was assessed using an eleven-item scale based on the foundational work of Eisenberger et al. (1986). This measure was specifically adapted into a Portuguese version to evaluate the degree to which individuals feel supported by their organization, both generally and contextually regarding technological change and AI implementation. Responses were captured on a 5-point Likert scale (1 = “Strongly disagree” to 5 = “Strongly agree”). A representative item utilized in this study is: “The organization strongly considers my goals and values when introducing AI technology in the workplace.” The scale demonstrated excellent internal metrics within the current sample (*α* = 0.89, CR = 0.90, AVE = 0.54).

Job security perceptions of Job Security were evaluated using a five-item scale originally developed by Hellgren et al. (1999), which was subsequently translated into Portuguese. Participants rated their agreement with statements concerning their sense of job stability and continuity using a 5-point Likert scale (1 = “Strongly disagree” to 5 = “Strongly agree”). An illustrative item adapted for the study is: “I am worried about having to leave my job due to AI before I would like to”. The scale demonstrated adequate psychometric fit ( α = 0.80, CR = 0.82, AVE = 0.51).

Career Competency: was assessed using a ten-item questionnaire developed by Kong et al. (2021), which was translated into Portuguese for the study. Respondents used a 5-point Likert scale (1 = “Strongly disagree” to 5 = “Strongly agree”). An illustrative item adapted for this study is: “I have the ability to learn from experience, especially in the context of AI-related tasks”. The instrument showed strong internal consistency ( α = 0.88, CR = 0.89, AVE = 0.52).

Career Satisfaction: was measured using a five-item scale developed by Greenhaus et al. (1990). Participants indicated their level of satisfaction with various aspects of their career using a 5-point Likert scale, ranging from 1 = “Strongly disagree” to 5 = “Strongly agree”. An example item is: “I am satisfied with the success I have achieved in my career, even with AI changing the nature of my work”. The scale met all validation benchmarks ( α = 0.88, CR = 0.89, AVE = 0.62).

The assessment protocol also included a set of open-ended questions designed to elucidate employees’ experiences and gather practical recommendations concerning AI integration in the workplace. The specific qualitative prompts utilized were:

1. In what ways do you think the organization could improve its support during the transition to AI?
2. What impact, if any, has AI had on your day-to-day responsibilities?

### Data collection and data analysis procedures

The research focused on the Portuguese context, with the target population defined as Portuguese employees working in AI-integrated workplaces. A non-probability sampling strategy, combining purposive and convenience sampling (through professional networks, LinkedIn, and company contacts), was used. Eligibility criteria included: (i) respondents had to be currently employed in an organization with AI integrated into business functions, and (ii) had to have direct or indirect exposure to AI technologies in their workplace.

Ethical guidelines were rigorously adhered to throughout the data collection period (late November 2024 to mid-March 2025). The survey design followed the ethical standards of the Catolica Research Centre for Psychological, Family and Social Wellbeing and complied with the General Data Protection Regulation (GDPR) in Portugal. Prior to responding, all participants were presented with an informed consent section detailing the study’s purpose, the voluntary nature of their participation, assurances of confidentiality and the right to withdraw at any time without penalty^1^. The average completion time for the full questionnaire ranged between 5 and 7 min.

The analysis began with a descriptive statistical overview of all key variables. To ensure the reliability and validity of the measurement, previously validated scales were used, and internal consistency was assessed using Cronbach’s Alpha (α), with values exceeding 0.70 considered acceptable (Nunnally, 1978).

To check for potential Common Method Bias (CMB) inherent in cross-sectional, self-reported data designs, Harman’s single-factor test was performed. All items (*n* = 35) from the five substantive scales were loaded into an unrotated exploratory factor analysis. The largest single factor accounted for 28.47% of the total variance, which is comfortably beneath the conservative 50% threshold benchmark, indicating that common method variance is unlikely to distort the interpretations of the statistical outcomes.

Hypotheses were tested in three main sequential phases utilizing IBM SPSS Statistics (v.30 for Windows) and Hayes’ PROCESS macro (Model 1): (i) a bivariate correlation analysis, to map baseline relationships, (ii) multiple hierarchical linear regression analyses incorporating control variables to test the main predictive power (Hypotheses H1 through H6) and, (iii) a moderation analysis utilizing mean-centered interaction terms to evaluate the buffering effects (Hypothesis H7).

Ordinary Least Squares (OLS) linear regression via Hayes’ PROCESS macro was selected over Structural Equation Modeling (SEM) due to sample size parameters. Given our final sample of *n* = 106, running a complex structural moderation model with multiple latent factors via SEM would likely result in parameter instability, insufficient statistical power, and non-convergence errors. OLS regression with interaction terms represents a mathematically robust, stable, and conservative alternative for this sample size context.

To ensure statistical transparency and account for sample limitations, an a posteriori statistical power analysis was calculated using G*Power for the multiple regression models. With an alpha level of α =0.05, a final sample size of *n* = 106, and three predictors, the analysis indicated that the statistical power was exceptionally high, exceeding 0.99 for detecting medium (*f*^2^ = 0.15 ) to large (*f*^2^ = 0.35 ) effect sizes. This confirms that the sample size provides more than adequate statistical power to reliably detect the core structural and moderation associations reported in this study.

In addition to the quantitative data, the survey incorporated two optional open-ended questions to generate rich qualitative insights regarding AI implementation in the workplace. A total of 68 participants provided valid responses to at least one of the two qualitative prompts. The participants’ responses to these questions should be considered as supplementary qualitative data rather than a full mixed-methods component. Specifically, 60 participants responded to the question “*Ways organizations can support employees during AI adoption*,” and 65 responded to the question “*Impact of AI on daily life.”* A significant overlap was observed, with 57 participants answering both questions, while 3 responded only to Question 39 and 8 responded only to Question 40.

The qualitative data were analyzed using Bardin’s (1977) content analysis framework. The process involved three distinct phases: (1) pre-analysis through floating reading of responses; (2) exploration of the material to define coding units; and (3) treatment and interpretation. To ensure analytical rigor, a multi-stage coding process was employed: Initial concepts were identified from the raw text (open coding); connections between these initial codes were established to form broader thematic categories (axial coding; e.g., connecting “organizational silence” with “increased job insecurity”); and, the final themes were integrated into a coherent narrative, grounded in the theoretical framework – namely in the Job Demands-Resources framework (selective coding). To ensure inter-rater reliability, two researchers conducted independent coding, resolving any discrepancies through consensus. An initial inter-rater agreement was calculated using Cohen’s Kappa (κ = 0.84), indicating a substantial level of agreement (Landis and Koch, 1977). Discrepancies were resolved through a consensus-seeking meeting until a 100% agreement was reached for the final coding scheme. This systematic approach facilitated the triangulation of the results with the participants’ lived experiences of AI integration.

## Results

### Quantitative results

This section details the findings from the statistical analyses performed to test the proposed theoretical framework. Table 1 presents the descriptive statistics and internal consistency measures of the variables under study. Participants indicated moderate degrees of AI-related insecurity (*M* = 2.18, *SD* = 0.96, *Mdn* = 2, *Mode* = 2), demonstrating a tendency towards prudence or ambivalence about the effects of AI. Conversely, scores on Perceived Organizational Support (*M* = 3.71, *SD* = 0.69, *Mdn* = 3.80, *Mode* = 4), as well as Job Security (*M* = 4.20, *SD* = 0.67, *Mdn* = 4.50, *Mode* = 5), were of a fairly high nature and indicate a safe and supportive work environment for employees. Similarly, both Career Competency (*M* = 3.72, *SD* = 0.50, *Mdn* = 3.67, *Mode* = 4), and Career Satisfaction (*M* = 3.81, *SD* = 0.73, *Mdn* = 4.00, *Mod*e = 4), were at high levels.

**Table 1:** Descriptive statistics and internal consistency estimates for main variables (*n* = 106).

Variable	M	Mdn	Mode	SD	Skew	Kurtosis	α (Cronbach)
AI – related insecurity	2.18	2.00	2	0.96	0.99	0.83	0.88
Organizational support	3.71	3.80	4	0.69	−0.20	−0.37	0.89
Job security	4.20	4.50	5	0.67	−1.13	2.06	0.80
Career competency	3.72	3.67	4	0.50	0.11	0.17	0.88
Career satisfaction	3.81	4.00	4	0.73	−0.07	−0.56	0.88

M = Mean. SD = Standard Deviation. α = Cronbach’s alpha; Skewness and kurtosis values indicate the shape of the distribution. Cronbach’s alpha values reflect internal consistency and reliability for each scale.

Bivariate Pearson correlations (see Table 2) were computed to investigate the linear correlations of all variables used in this study. AI-related insecurity holds a significant negative correlation with Job Security (*r* = −0.73, *p < 0*.001; thus supporting H1), Career Satisfaction (*r* = −0.22, *p <* 0.05; thus supporting H2), as well as with Perceived Organizational Support (*r* = −0.32, *p <* 0.01). These findings lend support to research hypotheses H1 and H2 and are consistent with the current literature pertaining to employee fears regarding the incorporation of AI. In contrast, the negative correlation held between AI-related insecurity and Career Competency (*r* = −0.18, *n.s.*; H3) failed to reach statistical significance and thus failed to support H1.

**Table 2:** Pearson correlation matrix between AI-related insecurity, career competency, career satisfaction, organizational support, and job security.

Variable	1	2	3	4	5
AI-related insecurity	—				
Organizational support	−0.18	—			
Job security	−0.22*	0.50**	—		
Career competency	−0.32**	0.50**	0.51**	—	
Career satisfaction	−0.73**	0.40**	0.42**	0.48**	—

*N* = 106. *p < 0.05, **p < 0.01. All tests are two-tailed.

Perceived Organizational Support (POS) also exhibited a significant and positive correlation with the three work outcomes: Career Satisfaction (*r* = 0.51, *p <* 0.001; H4c), Career Competency (*r* = 0.50, *p <* 0.001; H4b), and Job Security (*r* = 0.48, *p <* 0.001; H4a).

In addition, both Career Competency (*r* = 0.50, *p < 0*.001; H6), and Job Security (*r* = 0.42, *p <* 0.001; H5), were also positively and significantly correlated with Career Satisfaction and as such further established their positions as significant professional well-being drivers.

To guarantee that the structural relationships were not confounded by socio-demographic variations, all primary regression models were executed controlling for age, gender, and organizational tenure. These control covariates did not show a statistically significant effect on the primary endogenous outcomes, and their inclusion did not significantly alter the primary standardized regression paths, confirming that the structural model remains stable across demographic profiles.

Simple linear regressions were also conducted to analyze the directional hypotheses supplemented by 95% Confidence Intervals [CI] to establish parameter precision (see Table 3). Results showed that AI-related insecurity negatively predicted Job Security, *β* = −0.73, *t* (104) = −10.82, *p <* 0.001, 95% CI [−0.62, −0.42] accounting for 53% of the variance (*R^2^* = 0.53), thus providing good support for H1. Also, a moderate and statistically significant negative correlation was discovered between AI-related insecurity and Career Satisfaction (*β* = −0.22, *t* (104) = −2.33, *p =* 0.022, 95% CI [−0.31, −0.02], *R^2^* = 0.05), thus providing support for H2. Perceived Organizational Support (POS) emerged as a significant and positive predictor of all measured outcomes: Job Security (*β* = 0.48, *t* (104) = 5.59, *p <* 0.001, 95% CI [0.33, 0.60], *R^2^* = 0.23), confirming H4a; Career Competency (*β* = 0.50, *t*(104) = 5.91, *p <* 0.001, 95% CI [0.26, 0.47], *R^2^* = 0.25), confirming H4b; and, Career Satisfaction (*β* = 0.51, *t* (104) = 5.97, *p <* 0.001, 95% CI [0.36, 0.72], *R^2^* = 0.26), confirming H4c. Also, both Job Security (*β* = 0.42, *t* (104) = 4.67, *p <* 0.001, 95% CI [0.28, 0.64], *R^2^* = 0.17) and Career Competency (*β* = 0.50, *t* (104) = 5.86, *p <* 0.001, 95% CI [0.52, 0.94], *R^2^* = 0.25) were significant and positive predictors of Career Satisfaction, thus providing support for H5 and H6, respectively.

**Table 3:** Summary of simple linear regression analyses for hypotheses H1-H6.

Hypothesis	Predictor	Outcome	β	t	*p*	95% CI	*R^2^*	Support
H1	AI-related insecurity	Job security	−0.73	−10.82	<0.001	[−0.62, −0.42]	0.53	Yes
H2	AI-related insecurity	Career satisfaction	−0.22	−2.33	0.022	[−0.31, −0.02]	0.05	Yes
H3	AI-related insecurity	Career competency	−0.18	−1.83	0.070	[−0.19, 0.01]	0.03	No
H4a	Organizational support	Job security	0.48	5.59	<0.001	[0.33, 0.60]	0.23	Yes
H4b	Organizational support	Career competency	0.50	5.91	<0.001	[0.26, 0.47]	0.25	Yes
H4c	Organizational support	Career satisfaction	0.51	5.97	<0.001	[0.36, 0.72]	0.26	Yes
H5	Job security	Career satisfaction	0.42	4.67	<0.001	[0.28, 0.64]	0.17	Yes
H6	Career competency	Career satisfaction	0.50	5.86	<0.001	[0.52, 0.94]	0.25	Yes

Each model was tested separately using simple linear regression (*N* = 106). AI-related insecurity and Organizational Support were predictors across different models. All coefficients are standardized (β). Career Satisfaction was predicted in multiple models by different independent variables. *R^2^* = coefficient of determination. *p* < 0.05 was considered statistically significant.

To examine whether Perceived Organizational Support (POS) had a moderating role in the relationship between AI-related insecurity and two key dependent variables-Career Satisfaction and Job Security-two moderation analyses were carried out with mean-centered variables (see Table 4). For Career Satisfaction, the interaction effect between AI-related insecurity and POS was not statistically significant (*β* = −0.07, *p =* 0.298), thus indicating the absence of a moderation effect and failing to support H7c. The full model attained statistical significance, *F* (3,102) = 12.39, *p <* 0.001, explaining 27% of the variance (*R^2^* = 0.27). Together with the findings, it becomes clear that Perceived Organizational Support has a moderating role only in the relationship of AI-related insecurity and Job Security and has none with Career Satisfaction.

**Table 4:** Summary of moderation analyses: organizational support as a moderator (H7).

Outcome variable	β (interaction term)	*p*	*R^2^*	F(3, 102)	95% CI	Support
Career satisfaction	−0.07	0.298	0.27	12.39	[−0.17, 0.05]	No
Job security	0.10	0.033	0.62	54.43	[0.01, 0.19]	Yes

Each model was tested using PROCESS macro (model 1) with mean-centered variables (*N* = 106). β = standardized regression coefficient. *R^2^* = coefficient of determination. *p* < 0.05 was considered statistically significant.

However, for Job Security, the interaction term between AI-related insecurity and POS was statistically significant (β = 0.10, *p* = 0.033; 95% CI [0.01, 0.19]), providing support for H7a. This finding suggests that the moderating effect of POS on the AI-related insecurity and Job Security connection was significant. Specifically, the strong negative influence of AI-related insecurity on Job Security was weakened as the level of Perceived Organizational Support increased. The full model accounted for a substantial 62% of the variance (*R^2^* = 0.62) in Job Security, *F* (3,102) = 54.43, *p < 0*.001. These results collectively support the idea that Perceived Organizational Support has a moderating role only in the relationship of AI-related insecurity and Job Security, but not for Career Satisfaction.

To fully probe this significant interaction, a simple slopes analysis was conducted at ±1 SD from the mean of POS (Figure 2). When POS is low (−1 SD), AI-related insecurity severely and negatively predicts job security (*b* = −0.58, *t* = −8.11, *p* < 0.001, 95% CI [−0.72, −0.44]). However, when POS is high (+1 SD), the debilitating effect of AI insecurity on job security is significantly mitigated, though it remains functional and statistically significant (*b* = −0.38, *t* = −4.92, *p* < 0.001, 95% CI [−0.53, −0.23]).

**Figure 2:**
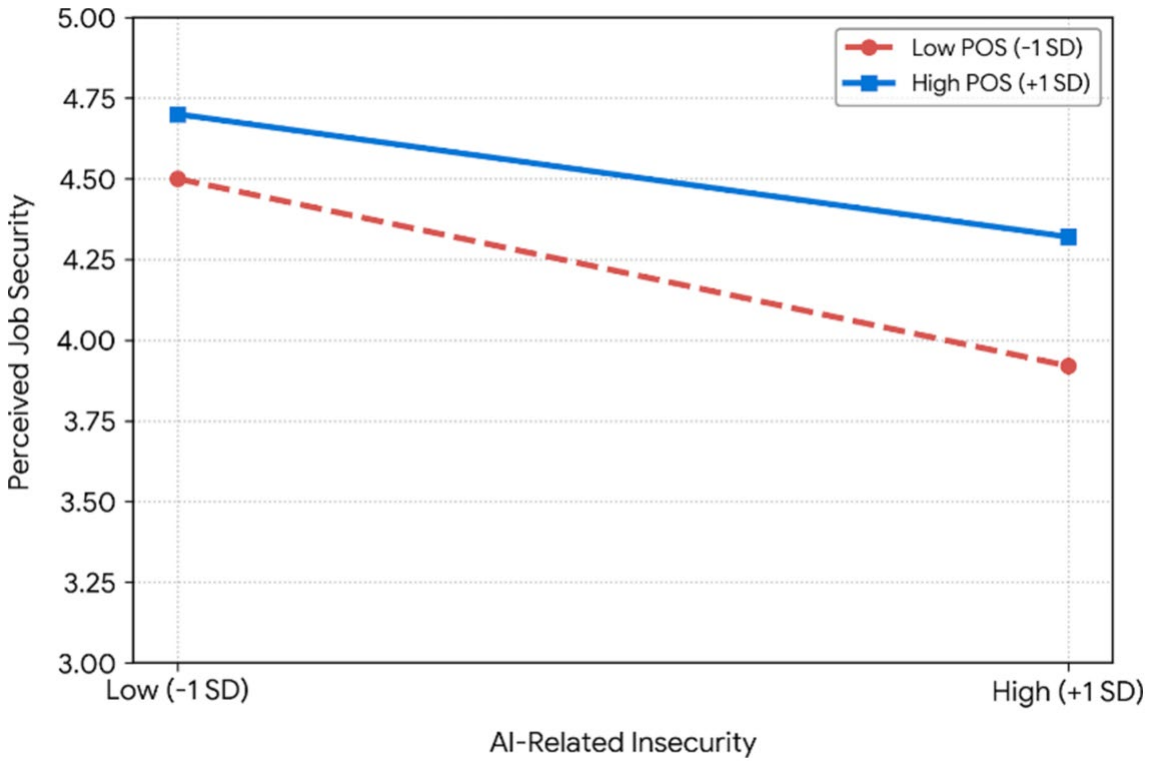
Moderation effect of perceived organizational Support on the relationship between AI-related insecurity and job security.

### Qualitative insights

This section presents the results of the qualitative analysis conducted on the participants’ responses to the two open-ended questions in the survey. The analytical process followed a systematic axial coding protocol, enabling the identification of patterns and the categorization of employees’ perceptions regarding the integration of Artificial Intelligence (AI) within their organizations. Table 5 systematizes this thematic mapping, presenting the axial categories, the identified subcategories, and direct qualitative evidence.

**Table 5:** Qualitative content analysis: axial coding and thematic mapping (*n* = 68) (the total of 75 coded references exceeds the number of participants (*n* = 68) as some respondents provided feedback covering multiple thematic categories).

Axial theme	Associated open codes (subcategories)	Representative verbatim quotes
Anxiety and uncertainty about the future of work (*n* = 28)	Job displacement; Devaluation of human labor; Professional identity threat	*“At the workforce level, I do not see this as a positive change… there could be a devaluation of labor, leading to potential layoffs”.* *“I feel that at any moment my role could be performed by a machine that is faster and cheaper.”*
Lack of clear and transparent communication (*n* = 16)	Organizational silence; Strategic ambiguity; Participatory implementation	*“Organizations should be clear and objective before any implementation… not use employees as lab rats without a prior strategy”.* *“Involving the team in decisions about AI integration can increase acceptance and build trust in the process.”*
Training and upskilling requirements (*n* = 22)	Proactive learning; Digital literacy gap	*“More proactive training is needed, rather than just reacting to external or internal organizational events”.* *“More specialized training. Regular and direct communication with employees is essential.”*
Believing in the augmentative power of AI (*n* = 9)	Efficiency gains; Decision support; Strategic time allocation	*“It allowed me to be faster in less complex tasks, enabling me to dedicate more time and deepen activities that require analytical depth”.* *“AI facilitates time-consuming tasks… streamlining decision-making and everything that it entails.”*

### Anxiety and uncertainty about the future of work

A leading theme arising from the open-ended responses concerned a deep sense of anxiety and uncertainty regarding the future of work amid the rising integration of artificial intelligence (AI). Many participants (*n* = 28) explicitly expressed concerns about potential displacement from work, declining value of human labor, and doubts over continuing professional utility in automated environments. This fundamental sentiment is encapsulated in illustrative responses *like “For the organizations, it seems to be beneficial, but for the workforce…”* and *“I am afraid this transition may lead to many firings”*. These thoughts not only indicate concern for the immediate impact of AI but also indicate apprehensions for the adequacy of organizational clarity and preparedness for managing workforce change. This anxiety was particularly prevalent among individuals with limited prior exposure to AI or those operating in sectors undergoing rapid technological transformation, such as education and finance. For these groups, concerns about obsolescence and ambiguity over changing professional obligations created the bulk of the psychological discomfort and doubts over the viability of a career.

### Lack of clear and transparent communication

Another significant theme emerging from the qualitative data was the expressed deficiency in transparent and consistent communication from organizations concerning the strategic incorporation of artificial intelligence (AI) technologies. A few respondents (*n* = 16) reported a lack of adequate information or felt excluded from dialogues pertaining to their organization’s AI strategies, implementation schedules, and objectives. Representative comments, such as *“We hear rumors about AI coming, but no one really explains how it will affect our jobs”,* and *“There needs to be more regular and direct communication about AI”,* highlight that inadequate internal communication exacerbates uncertainty, fosters mistrust, and engenders confusion among employees.

### Training and upskilling requirements

Twenty-two of the respondents emphasized the need for structured training and upskilling programs due to the increasing prevalence of the use of artificial intelligence (AI) tools and systems at the workplace. Several workers reported a lack of preparedness for managing the impacts of change driven by AI and a lack of confidence when implementing new technologies. Statements such as *“Organizations need a better and more efficient adaptation,”* and *“I am not sure I’ll be able to keep up without proper guidance”,* reflect a perceived competency gap. These remarks demonstrate a growing awareness among employees that their existing skill sets may not suffice in an increasingly automated and technologically integrated professional landscape. Beyond articulating concern, respondents explicitly called for dedicated, structured learning opportunities-ranging from workshops and tutorials to mentoring and ongoing digital literacy programs. Comments like *“We need reskilling programs, not just talks about AI”,* and *“More workshops in how to use these tools”,* suggest that employees are not resistant to change *per se* but are actively seeking the necessary tools and support to navigate the transition successfully.

### Believing in the augmentative power of AI

A minority of the respondents (*n* = 9) reported a constructive and optimistic perspective of combining artificial intelligence (AI). The respondents insisted that, when used carefully, AI would complement the abilities of humans, enhance productivity, reduce the potential for routine work, and facilitate general improvement of work quality and efficiency. The respondents recognized the capability of artificial intelligence (AI) for enhancing work processes significantly but in positive and substantive ways. Comments such as *“AI helps me with time management”,* and *“It helps me improve writing emails, especially when I have mental blocks”,* indicate that they envisioned AI as a complementary means for human labor, also with the potential for enhancing innovation, supporting professional growth, as well as enabling creative or high-value inputs. This optimistic view frequently came with the condition for the availability of easily accessible training facilities, which instilled a sense of preparedness and portrayed the adoption of AI as a potential opportunity for mutual advancement.

## Discussion

The adoption of AI in the workplace has become an increasing and irreversible global trend. This study aimed to understand how AI adoption affects employees’ perceptions and adaptation within the Portuguese organizational context and whether Perceived Organizational Support (POS) can exert a buffering effect.

The results obtained are, in general, congruent with the growing literature in the field, highlighting the significant and expected repercussions of integrating artificial intelligence systems into daily work practices. These repercussions translate into an amplification of AI-associated anxiety and fears, particularly related to job displacement and the diminishing relevance of existing skills (Acemoglu and Restrepo, 2018; Sadeghi, 2024; Zhou et al., 2024).

The quantitative finding that AI-related insecurity significantly and negatively predicts Job Security (H1: supported) and Career Satisfaction (H2: supported) is a direct empirical reflection of this psychological tension. This is strongly corroborated by the qualitative insights, which show prevalent anxiety and uncertainty regarding job displacement. Interestingly, the predictive relationship with Career Competency (H3) was not statistically confirmed (though directionally negative), suggesting that while employees fear the structural impact of AI on their job stability and overall satisfaction, they may maintain a baseline confidence in their current, adaptable skills. The negative psychological impact of this AI-related insecurity is not exclusive to the Portuguese context, being a globally recognized challenge (e.g., Elfar, 2025). Therefore, for AI integration to be sustainable in the long term and avoid causing harm, it must be accompanied by proactive and strategic efforts to address employees’ social and psychological well-being (Budhwar et al., 2022; Makarius et al., 2020).

This observation is reinforced by qualitative evidence, in which participants frequently described artificial intelligence not as a threat, but rather as an empowering tool for process streamlining and value-enhancing task enablement. Objective data support this context: Portugal is in a strong position regarding AI readiness, particularly in terms of policy safety (Oxford Insights, 2024), and domestic initiatives are already underway to boost skill levels among a substantial portion of the workforce (McKinsey Global Institutute, 2024). These results suggest that Portugal can constitute a relevant comparative context for other nations seeking to balance technological incorporation with employee well-being and skill flexibility. Nevertheless, several issues persist in terms of addressing structural and strategic weaknesses in AI governance.

### The asymmetric buffering phenomenon

Results also provide robust confirmation for the idea that fostering a strong support system is essential for managing the human implications of technological disruption. Specifically, the finding that Perceived Organizational Support (POS) positively predicts Job Security, Career Competency, and Career Satisfaction (H4: supported, with H4a, H4b, and H4c all supported) is fundamental. Furthermore, POS acts as a critical psychological safety net, mitigating job insecurity and reinforcing employee confidence, as evidenced by the significant moderation effect found between AI-related insecurity and Job Security (H7a: supported). This is highly congruent with the Job Demands-Resources (JD-R) Theory (Bakker and Demerouti, 2017), framing POS as a core resource to effectively reduce the costs associated with the demands of technological uncertainty. Qualitative results also support this effect, with participants explicitly urging for strategic training, *upskilling*, emotional support, and organizational transparency to compensate for the uncertainty caused by artificial intelligence (Al Nahyan et al., 2024; Shantz et al., 2016; Zhou et al., 2023). Thus, organizational support is instrumental in fostering psychological resilience in the modern technology-driven workplace.

These results also reinforce the predictive importance of Job Security (H5: supported) and Career Competency (H6: supported) as critical drivers of Career Satisfaction. The strong empirical support for both hypotheses reinforces the need for a dual-pronged organizational strategy, focusing both on the hygiene factor of stability (Job Security) and the motivational factor of growth (Career Competency), according to Herzberg’s Two-Factor Theory (Herzberg, 1975). The convergence of these factors is, in fact, central to Smerek and Peterson’s (2006) Assessment Model of Job Satisfaction. Lastly, POS enables these positive outcomes by ensuring the internal conditions necessary for employee development across multiple fronts/ dimensions, fostering mutual trust and commitment.

However, despite Perceived Organizational Support (POS) being central to organizational well-being, it does not emerge as a one-size-fits-all solution. This is indicated by the non-significance of POS moderation between AI-related insecurity and Career Satisfaction (H7c: not supported) and Career Competency (H7b: not supported). The lack of moderation across all outcomes suggests that simple social or emotional support may be insufficient to address deeper, structural concerns, such as long-term role understanding and mobility opportunities (Eisenberger et al., 2019; Wang and Wang, 2020; Xu and Yang, 2018).

This result introduces a critical tension into classic literature, compelling to a reassessment of fundamental assumptions within mainstream models, such as the Job Demand-Control-Support (JDCS) framework (Kain and Jex, 2010) and the Conservation of Resources (COR) Theory (Hobfoll, 2010) regarding the sufficiency and scope of organizational resources. The complexity of this result is further highlighted by the potential for “reverse-buffering” (Pharris et al., 2025; Semmer et al., 2025)—where poorly timed or misaligned support can heighten, rather than alleviate, employee stress (Gray et al., 2022).

The qualitative theme of deficiencies in clear and transparent communication provides a clear context for why support might fail (H7c, H7b): if employees feel excluded from strategic discussions, no amount of generic support will mitigate their role uncertainty. This suggests that when communication is perceived as opaque, general POS is interpreted by employees as a reactive measure rather than a proactive strategic commitment. Consequently, the absence of clear, strategic information acts as a ‘resource drain’ that neutralizes the potential buffering effect of POS on career satisfaction. In other words, generic support fails to protect long-term career wellbeing because it does not address the core uncertainty identified in our qualitative analysis: the need for a concrete and communicated roadmap for human-AI collaboration. Organizations must, therefore, be intentional not only about what support they offer, but also about how and when that support is delivered.

### Sociotechnical and ethical repercussions

In summary, these findings highlight a critical gap between organizational policies and employees’ perceptions. Despite potential internal initiatives aimed at reassuring employees, the risks associated with AI are consistently not perceived as adequately addressed, indicating a sense of vulnerability and distrust among the workforce (Sadeghi, 2024; Soulami et al., 2024; Vuori et al., 2025). This gap highlights the need for intentional and systematic change management methods, coupled with a broader policy debate about the ethical incorporation of AI. It also underscores the demand for human impact analyses and a commitment to psychological safety throughout the entire adoption framework (Ayling and Chapman, 2021; Stahl et al., 2021; Zawacki-Richter et al., 2019).

The most recent literature supports the view that psychological harm can be caused if artificial intelligence systems erode professional judgment or dignity (Cebulla et al., 2022; Salvi del Pero et al., 2022). By accepting this reality, organizations can bring AI ethics into the debate as a workplace health and safety issue that is critical for mediating sustainable and rapid technological adoption.

### Practical implications for organizations

The empirical insights yield concrete managerial and HR recommendations for organizations undergoing digital transformations. First, because generalized emotional support fails to safeguard long-term career satisfaction, organizations must move away from generic reassurance strategies. HR departments should implement transparent, dual-channel communication protocols that outline explicit human-AI collaboration roadmaps, ensuring employees are structurally included in technological procurement choices.

Second, companies must establish formal reskilling and continuous digital literacy programs that target qualitative skill obsolescence. Rather than focusing solely on basic tool operations, training should empower professionals to build advanced complementary competencies, such as data interpretation and human-in-the-loop validation, thereby reinforcing their long-term professional marketability.

Lastly, tech adoption frameworks must undergo explicit psychological safety risk audits, treating AI integration not as a pure software deployment, but as an ethical sociotechnical intervention that directly impacts workforce mental health.

### Methodological limitations and future lines of research

Despite the novel contributions, the current study is subject to several limitations that must be acknowledged. First, due to the cross-sectional nature of data collection, concerns regarding Common Method Bias (CMB) and reverse causality must be addressed. All variables were collected via self-report in a single survey, which may inflate correlations. While we employed validated scales with high internal consistency (all α > 0.80) and confirmed via Harman’s test that common variance did not dominate the dataset, these findings should be interpreted as associations rather than established causal links. Future longitudinal research is needed to confirm the directionality of the predicted relationships.

Second, the sample size (*n* = 106) may limit the statistical power required to detect complex moderation effects for Career Satisfaction and Competency. Furthermore, the reliance on convenience and purposive sampling via digital professional networks generates potential self-selection biases, as individuals with strong personal views on AI might have been more prone to complete the instrument.

Additionally, the sample’s profile-predominantly female (69.8%) and highly educated (85.8% with a degree)-limits the generalizability of our findings to less specialized sectors or populations with different educational backgrounds. The single-country setting also restricts direct translation to non-European corporate cultures. Future studies should prioritize broader, multi-national sampling methods and structural model replications to fully validate the cross-cultural boundary parameters of the ‘Asymmetric Buffering’ phenomenon.

Third, the adopted approach is primarily quantitative; while we included insights from open-ended questions, a true mixed-methods approach (e.g., in-depth interviews) would allow for a richer and more contextualized understanding of the experience of AI-related insecurity in the workplace.

## Conclusion

The proliferation of Artificial Intelligence (AI) constitutes an undeniable force reshaping organizational dynamics, making the study of its human impact critical. This paper investigated the role of Perceived Organizational Support (POS) as both a predictor and a mitigating factor against employee outcomes-specifically Job Security, Career Competency, and Career Satisfaction-amidst AI integration within the Portuguese context.

Quantitative analysis confirmed that employee AI-related insecurity significantly and negatively predicted both Job Security and Career Satisfaction. Conversely, POS emerged as a robust positive predictor across all three employee outcomes. The most pivotal finding of this study is the demonstration of an asymmetric buffering response. Although Perceived Organizational Support (POS) is an effective antidote against the proximate, concrete threat of job loss (Job Security), POS alone is not effective at buffering against the intangible, strategic fears relating to the employee’s broader career path (Career Satisfaction and Competency). This result challenges a unitary application of the JD-R theory’s buffering hypothesis, suggesting that organizational resources may only successfully neutralize immediate, “hygiene” demands (like job security) but fail to mitigate deeper, strategic “motivational” demands concerning long-term career fulfillment.

Complementary qualitative findings highlight a profound policy-perception gap. Widespread employee anxiety is stimulated by obsolescence fear and strong encouragement for proactive communications and strategic upskill. While lending support to the contention that POS operates as a buffer, results stress that support needs to align strategically with technological deployment. Poorly designed support, as noted in the literature on reverse buffering, risks exacerbating employee distress.

Ultimately, this study contributes to the digital transformation discourse by confirming that AI-related insecurity poses a palpable threat to employee stability and well-being. It concurrently illustrates that meticulously adjusted POS functions as an essential, albeit incomplete, alleviating element.

For the future research agenda, we strongly suggest making use of longitudinal and mixed-method designs to better reflect the changing complexity of integrating AI. Beyond this, the Portuguese context, with what appears to be a more restrained approach to adopting AI, provides some strong ground for cross-cultural comparative research contrasting with results from more enthusiastically and (aggressively) automated economies.

## Data Availability

The raw data supporting the conclusions of this article will be made available by the authors, without undue reservation.

## Footnotes

1
https://ucpcienciashumanas.eu.qualtrics.com/jfe/form/SV_87U4eRy4iN31FX0
